# Fine needle aspiration cytology of lymph nodes in breast cancer follow-up is a feasible alternative to watchful waiting and to histology

**DOI:** 10.1186/s12905-015-0269-z

**Published:** 2015-12-03

**Authors:** Matthias Hammon, Peter Dankerl, Rolf Janka, David L. Wachter, Arndt Hartmann, Rüdiger Schulz-Wendtland, Michael Uder, Evelyn Wenkel

**Affiliations:** Department of Radiology, University Hospital Erlangen, Maximiliansplatz 1, 91054 Erlangen, Germany; Department of Pathology, University Hospital Erlangen, Erlangen, Germany

**Keywords:** Lymphatic, Metastasis, Breast, Ultrasound, Fine-needle aspiration cytology

## Abstract

**Background:**

Early detection of loco-regional breast cancer recurrence improves patients’ overall survival, as treatment can be initiated or active treatment can be changed. If a suspicious lymph node is diagnosed during a follow-up exam, surgical excision is often performed. The aim of this study was to evaluate the diagnostic performance of the minor invasive ultrasound-guided fine-needle aspiration cytology (FNAC) in sonomorphologically suspicious lymph nodes in breast cancer follow-up.

**Methods:**

Between April 2010 and November 2012, we performed ultrasound-guided FNAC in 38 sonographically suspicious lymph nodes of 37 breast cancer follow-up patients. Cytological specimens were evaluated if the sample material was sufficient for diagnosis and if they contained cancer cells. Patients with negative cytology were followed up clinically and sonographically. To evaluate the diagnostic performance we calculated sensitivity, specificity, positive predictive value (PPV) and negative predictive value (NPV) for physical examination, the different sonomorphological malignancy criteria and FNAC.

**Results:**

In 36/38 (94.7 %) lymph nodes, the pathologist had enough material to establish a final diagnosis; in 2/38 (5.3 %) lymph nodes, the probe material was non-evaluable during cytology, these 2 were excluded from further statistical evaluation. Cytology revealed malignancy in 21 lymph nodes and showed no evidence for malignancy in 15 lymph nodes. There was no evidence for malignant disease in follow-up exams in the 15 cytologically benign lymph nodes with an average follow-up time of 3 years. The diagnostic performances of physical examination and FNAC were: Sensitivity 52/100 %, specificity 88/100 %, PPV 85/100 %, NPV 60/100 %, respectively.

**Conclusions:**

Our preliminary results show that FNAC is a safe and fast diagnostic approach for the evaluation of suspicious lymph nodes in the follow-up of patients with breast cancer and, thus, together with follow-up represents a feasible alternative to surgery.

## Background

Breast cancer is the most frequently diagnosed solid cancer in women and one of the leading causes of cancer deaths in the western world [1]. While screening mammography has led to the earlier detection of breast cancer [2], and guideline adherent therapy has improved overall and recurrence-free survival [3], detection of breast cancer recurrence remains difficult [4]. Data from meta-analysis and retrospective studies confirm that early detection of local recurrences resulted in significantly better survival as compared to late detected recurrences [5–7].

Regional lymph node recurrence is uncommon and has been reported in 1–3 % of patients with early stage breast cancer and in 1.7–15.9 % of patients with any stage of breast cancer [8]. The examinations performed to detect and assess lymph node recurrence should be reasonable regarding the patient’s quality of life and time and cost-effectiveness [9]. Follow-up exams have to manage the difficult task of not inducing too much anxiety in patient with unnecessary exams or interventions and to minimize complications and costs [10].

Loco-regional recurrence includes recurrent disease in the diseased breast and the ipsilateral lymph nodes in the axillary, the supra- and infraclavicular and the internal mammary region. Regular follow-up exams are dependent on institutional preferences and include physical examination, mammography and sonography and, in unclear breast findings, magnetic resonance tomography [11, 12]. In newly diagnosed breast cancer, axillary lymph node evaluation is usually performed preoperatively by clinical examination, sonography and lymph node resection. However, core needle biopsy and ultrasound-guided fine-needle aspiration cytology (FNAC) [13, 14] have become possible alternatives. In newly diagnosed breast cancer, it has already been shown that FNAC can achieve high accuracy, sensitivity and specificity [11, 15–20] to predict lymph node metastases. To the authors’ knowledge, there are no prospective studies about lymph node sonography and FNAC in follow-up exams of breast cancer patients.

The aim of our study was to evaluate the diagnostic performance of fine-needle aspiration cytology of sonomorphologically suspicious lymph nodes in breast cancer follow-up.

## Methods

### Ethics statement

This single-centre investigation was approved by the institutional review board of the University Hospital Erlangen and all procedures were in accordance with the Helsinki Declaration. The need for informed consent was waived.

### Patients

From April 2010 to November 2012, we performed more than 2500 follow-up exams in patients with breast cancer. Routine follow-up of patients included clinical examination and sonography of the breasts and the locoregional lymph node stations every 6 months for the first 3 years after surgery and then in yearly intervals. Mammography of the affected side was performed every 6 months for the first 3 years after surgery and then yearly; the non-affected breast was examined by mammography yearly. Patients who presented with sonomorphologically suspicious lymph nodes in the ipsi- or contralateral axillary or supra/infraclavicular region were eligible to receive FNAC. Prior to ultrasound-guided FNAC, written informed consent was obtained from each patient. During this time frame we performed ultrasound-guided FNAC in 59 sonographically suspicious lymph nodes of 58 patients. Of these, 38 FNAC probes from 37 patients were obtained from breast cancer follow-up patients and enrolled for further investigation. The remaining 21 suspicious lymph nodes of 21 patients were examined for non-breast cancer follow-up reasons and therefore not included in this study. Patients with positive cytology received recurrent stage adapted treatment. All patients with negative FNAC results were to follow up by sonography of the locoregional lymph nodes stations with special focus on the initially punctured lymph node within three months after the FNAC. If this evaluation revealed no change and no further suspicious findings, these patients should return to their regular follow-up examinations.

### Sonography

**Fig. 1:**
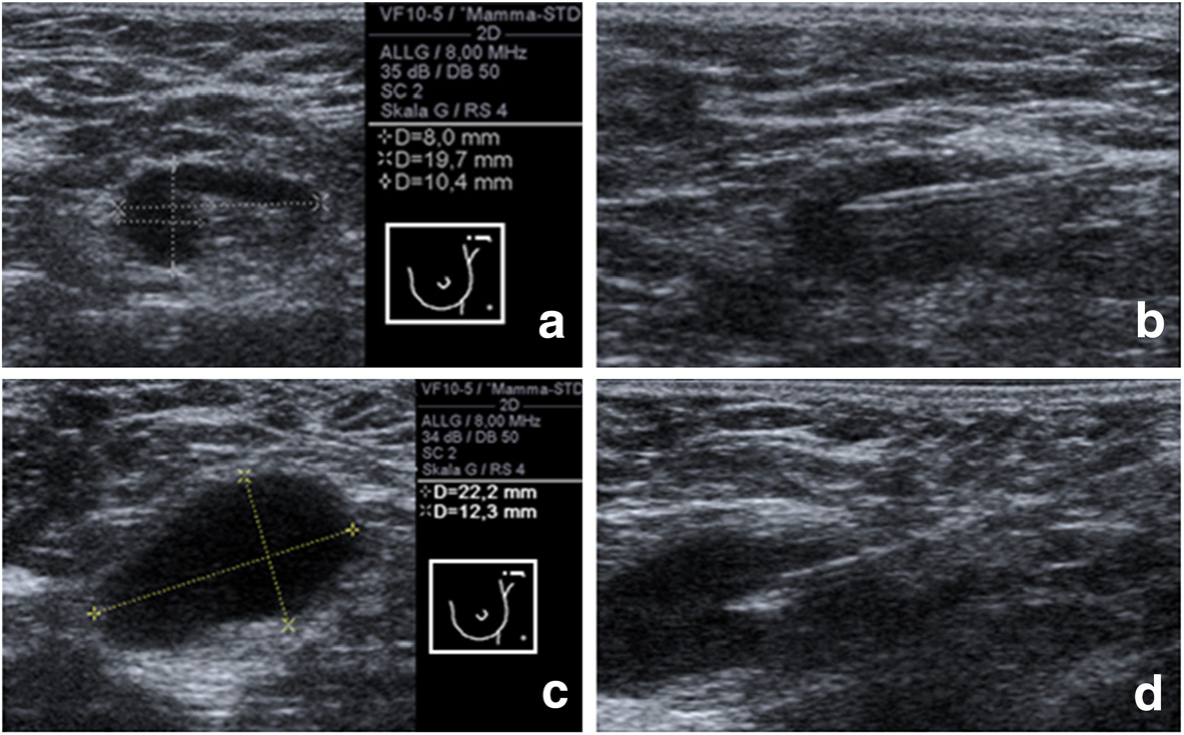
Examples of sonographically suspicious axillary lymph nodes that received further fine needle aspiration cytology and cytological evaluation. **a** Lymph node presenting a normal size, a longitudinal/transversal ratio < 2, a focal thickening of the cortex (8 mm) and an eccentric hilum. **b** Ultrasound-guided fine-needle aspiration cytology. **c** Lymph node with a round shape (longitudinal/transversal ratio < 2), an indeterminate size and loss of fatty hilum. **d** Ultrasound-guided fine-needle aspiration cytology

Sonography was performed by a diagnostic breast specialist (E.W.) with 10 years of experience or by supervised residents experienced in breast diagnostic rotation with high-resolution sonography equipment (15 MHz, electronic focus, linear-array transducer) (Siemens Acuson, Erlangen, Germany). A lymph node was defined as sonographically suspicious if one of the following characteristics was positive: size (longitudinal > 2 cm; transverse > 1.5 cm); shape (longitudinal/transversal ratio < 2, round), loss of central fatty hilum, cortex (eccentric or thickened; > 3 mm, as adopted by Oz et al. [17]) (Fig. 1). Additionally, all sonographically suspicious lymph nodes were measured in two planes. If one patient presented with more than one pathological lymph node in the same lymph drainage region, the most suspicious lymph node was selected for FNAC. If the suspicious nodes were in different topographical regions, one suspicious node in every region could receive FNAC.

### Fine-needle aspiration cytology (FNAC)

All ultrasound-guided FNAC were performed by one breast specialist (E.W.). After sterile draping and sufficient disinfection, a 22-gauge needle attached to a 10-ml syringe was inserted into and withdrawn from the lymph node three times under aspiration while sonographically monitored. For suspiciously enlarged or shaped lymph nodes, the needle was directed into multiple areas of the cortex and/or hilum. For irregularly shaped lymph nodes, the needle was directed into the thickest or focally thickened area of the cortex and/or hilum (Fig. 1). Succeeding FNAC, the probe was sent to cytology. Following FNAC, patients were evaluated clinically for 60 min and 1 week after the procedure. Complications (bleeding, hematoma, infection, loss/increased sensation) were also documented.

### Cytological evaluation

**Fig. 2:**
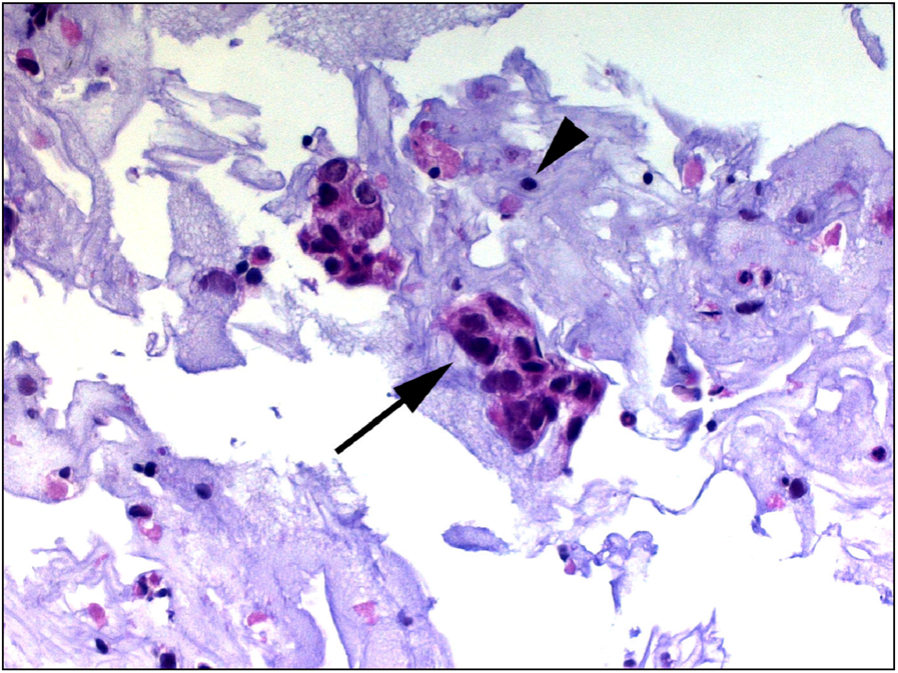
Cell block cytology of an investigated lymph node displaying poorly differentiated breast cancer cells (arrow). The arrowhead indicates a lymphocyte

An expert in cytology and breast pathology [D.W.] with 9 years of experience processed and evaluated all FNAC material. In order to be able to perform immunocytochemical examinations in equivocal cases, we used cytocentrifugation and the cytoblock technique [21]. The FNAC cytology sample was concentrated by centrifugation (600 g for 10 min). The supernatant was removed, and the pellet was resuspended in PreservCyt Solution (Cytyc Corporation, Marlborough, MA, USA). After sitting for 15 min, the sample was run on a ThinPrep 2000 Processor (Cytyc Corporation, Marlborough, MassachusettsMA, USA). The resulting slide was stained with Papanicolaou stain, covered with a coverslip, and evaluated. For the cytoblock technique, part of the supernatant was fixed in 4 % formaldehyde overnight. The next day the specimen was mixed with Histogel (Thermo Scientific, Waltham, MA, USA) and was allowed to harden. Subsequently the cytoblock was cut in 3 μm slices and the slides were stained in Hematoxylin/Eosin. Specimens were interpreted as follows: insufficient, no cellular material other than blood; negative, only normal cellular components present or a specific benign diagnosis; suspicious or malignant, atypical cells suspicious or definite for malignancy present (Fig. 2).

### Statistical analysis

Statistical analysis was performed using SPSS software version 21 (IBM, Armonk, New York, USA). Sensitivity, specificity, negative- (NPV) and positive-predictive value (PPV) were evaluated for physical examination, for the different sonomorphological malignancy criteria and for FNAC.

## Results

### Study population

From April 2010 to November 2012, a total of 57 women and 1 man underwent ultrasound-guided fine-needle aspiration cytology of 59 axillary or supra- and infraclavicular lymph nodes. A total of 21 women with 21 cytologically evaluated lymph nodes were excluded because they were not breast cancer follow-up patients. Those women received FNAC for other reasons, like primary staging of breast cancer (*n* = 9), staging of other malignancies (*n* = 5) or primarily unknown reasons for lymph node enlargement (*n* = 7; mastitis, infection). Therefore, FNAC was performed on 38 lymph nodes in 37 follow-up patients for breast cancer. The 37 patients consisted of 36 women and one man with a mean age of 57.0 ± 12.8 years. A total of 19 FNAC were taken in the ipsilateral axilla, 14 in the contralateral axilla, 2 in the ipsilateral supraclavicular region, 1 in the contralateral supraclavicular region, 2 in the ipsilateral infraclavicular region and none in the contralateral infraclavicular region (Table 1). Follow-up time from primary diagnosis of breast cancer to the diagnosis of a sonomorphologically suspicious lymph node ranged from one month to 26 years and 6 months (Table 1).

**Table 1:** Lymph node and patient characteristics dependent on the fine-needle aspiration cytology result

	Malignant	Benign	Non-evaluable
Lymph nodes (*n* = 38 in 37 patients)	21	15	2
Mean age in years (range)	57 (38 – 72)	56 (30 – 82)	65 (65 – 65)
Lymph node palpable in clinical exam	11	2	0
Mean interval from primary diagnosis to ultrasound-guided fine-needle aspiration cytology (range)	4y9m (1 m - 13y7m)	7y5m (5 m - 26y6m)	4y6m (2y3m - 6y9m)
Lymph node localisation			
• axillar ipsilateral	12	6	1
• axillar contralateral	7	6	1
• supraclavicular ipsilateral	1	1	0
• supraclavicular contralateral	1	0	0
• infraclavicular ipsilateral	0	2	0
• infraclavicular contralateral	0	0	0

### Physical examination

A total of 13/38 sonographically suspicious lymph nodes were additionally identified by palpation, of which 7 were located in the ipsilateral axilla, 5 in the contralateral axilla and 1 in the contralateral supraclavicular. A total of 11 of these 13 lymph nodes demonstrated a malignant cytology in FNAC. A total of 10 metastatic lymph nodes were not suspicious upon palpation, of which 6 were located in the ipsilateral and 4 in the contralateral axilla. This corresponds to a sensitivity of 52.4 % regarding palpation, a specificity of 88.2 %, a positive predictive value (PPV) of 84.6 % and a negative predictive value (NPV) of 60 %.

### Sonography

Sonomorphological characteristics of the lymph nodes, which were further assessed with fine-needle aspiration cytology, are shown in Table 2. All 59 lymph nodes showed at least one sonomophological pathological feature. The diagnostic performance of each individual sonomorphological malignancy criterion for lymph node evaluation is depicted in Table 3. The sensitivity and the specificity of the different malignancy criteria ranged from 19–71 % and from 29–88 %, the PPV from 42–75 % and the NPV from 39–57 %.

**Table 2:** Sonomorphological characteristics of the lymph nodes which were further assessed with fine-needle aspiration cytology

	Metastasis (*n* = 21)	No metastasis (*n* = 15)	Non-evaluable cytological probe (*n* = 2)
Lymph node size longitudinal			
> 2.0 cm	15	8	1
< 2.0 cm	6	7	1
Lymph node size transversal			
> 1.5 cm	6	2	0
< 1.5 cm	15	13	2
Lymph node shape (longitudinal/transversal ratio)			
- round (<2)	14	13	0
- oval (>2)	7	2	2
Hilum			
- loss of fat	16	10	2
- eccentric	4	2	0
Thickened cortex	5	6	1

**Table 3:** Diagnostic performance of physical examination, different sonomorphological malignancy criteria and fine-needle aspiration cytology for lymph node evaluation in breast cancer follow-up

	Sensitivity	Specificity	Positive predictive value	Negative predictive value
Physical examination	52.4 %	88.2 %	84.6 %	60 %
Sonomorphological malignancy criteria				
Lymph node enlargement:				
- longitudinal > 2.0 cm	71.4 %	47.1 %	62.5 %	57.1 %
- transversal > 1.5 cm	28.6 %	88.2 %	75 %	50 %
Round shape (longitudinal/transversal ratio <2)	66.7 %	23.5 %	51.8 %	36.4 %
Loss of fatty hilum	76.2 %	29.4 %	57.1 %	50 %
Eccentric hilum	19 %	88.2 %	66.7 %	46.9 %
Thickened cortex	23.8 %	58.8 %	41.7 %	38.5 %
Fine-needle aspiration cytology^a^	100 %	100 %	100 %	100 %

^a^excluding the two non-evaluable cytological probes

### Fine-needle aspiration cytology and clinical consequences

FNAC evaluation revealed metastases in 21 lymph nodes in 21 patients, 20 women and 1 man. A total of 16 patients with positive FNAC received surgical resection of the suspicious lymph nodes. Specimen pathology confirmed malignancy in all of these cases. A total of 5 patients with positive cytology did not undergo surgery, and were instead treated with chemotherapy, antihormonal therapy and/or radiation therapy.

Probe material gathered by fine-needle aspiration was non-evaluable during cytology in 2 lymph nodes (5.3 %) of two different patients because insufficient cell material was obtained. One was located in the ipsilateral and one in the contralateral axilla. Both patients with non-diagnostic cytological findings received successive lymph node resection of the initially biopsied lymph nodes. Specimen pathology did not reveal malignancy in either lymph node. FNAC did not indicate malignancy in 15 lymph nodes from 14 patients (Table 1).

The follow-up group consisted of 16 patients with 17 evaluated lymph nodes including the ones with negative cytology and the 2 patients with benign results after resection. To date, none of these patients presented with lymph node recurrence in the cytologically evaluated sites. The follow-up time of the patients with negative cytology or histology ranged from 22 months (1 year 10 months) to 53 months (4 years 5 months). Thus, FNAC demonstrated sensitivity and specificity of 100 % of sonomorphologically suspicious lymph nodes excluding the two cytologically non-evaluable probes.

### Fine-needle aspiration cytology side-effects

None of our patients demonstrated acute or delayed side effects from FNAC (bleeding, hematoma, infection, loss/increased sensation).

## Discussion

Follow-up exams in breast cancer patients have to manage a fine line between early detection of recurrence and overdiagnosis. Follow-up concepts include clinical examination and mammography [11, 22, 23]. Lymph node sonography is performed in most institutions only if lymph node stations are suspicious on palpation or more uncommonly on each follow-up appointment [11, 12, 23, 24]. MRI is usually recommended if the differentiation between scar and recurrent disease is necessary or in women at high risk for breast cancer [25]. If there is an unclear lymph node finding on clinical or imaging examination, the woman has the choice between invasive diagnostic procedures like core needle biopsy or lymph node dissection. On the other hand, the woman could choose short follow-up exams especially in unclear lymph node findings. This might induce unnecessary anxiety in the patient up to the next control date.

The minimally invasive procedures core needle biopsy [13, 26, 27] and FNAC have already been successfully tested in lymph nodes of newly diagnosed breast cancer [13, 14, 20, 28–31]. For core needle biopsies, a technique adapted to the axillary anatomy is recommended to minimize side effects [26]. A meta-analysis of data from 21 studies and 4313 patients showed that the pre-operative ultrasound of axillary lymph nodes in primary breast cancer resulted in a median sensitivity of 61.4 % (interquartile range 51.2–79.4 %) and a median specificity of 82 % (interquartile range 76.9–89 %) [32]. In a subset of 1733 patients in the same meta-analysis, the median sensitivity of ultrasound needle biopsy (results for core biopsy and FNAC) was 79.4 % (interquartile range 68.3–88.9 %) and the median specificity was 100 % (interquartile range 100–100 %) [32]. No explicit difference was found in this meta-analysis whether FNAC or core biopsies were performed. In a meta-analysis of 20 studies with 1371 subjects, the performance of FNAC for lymph node metastases in primary breast cancers resulted in a pooled sensitivity of 66 % (95 % CI was 64–69 %) and a specificity of 98 % (95 % CI was 98–99 %) [14]. As presented the diagnostic performance, especially the sensitivity of FNAC in post-operative breast cancer follow-up is much higher than in the preoperative staging of breast cancer patients. We believe that this is due to the different workflows and evaluation processes. In preoperative staging FNAC has been performed even if no lymph node was sonomorphologically suspicious on a random lymph node, in order to rule out metastases. However, if micrometastases had been present and coincidentally FNAC had been performed on a lymph node from a different draining system, these metastases could have not been detected by FNAC. The by us presented workflow eliminates the uncertainty of which lymph node to pick for FNAC, as the probed lymph node had to be newly detected and suspicious (either by physical examination or sonography), possibly explaining the perfect sensitivity. The excellent specificity of FNAC is in accordance to previous work [14], especially when performed by an experienced cytologist.

As in the pre-surgical studies, we show that FNAC is also reliable in lymph node stations that have already been tested. FNAC demonstrated sensitivity and specificity of 100 % during the assessment of sonomorphologically suspicious lymph nodes when excluding two cytologically non-evaluable probes. Regarding cost-effectiveness, FNAC is proposed to be routinely included in pre-operative evaluation of lymph nodes [10, 13, 32, 33]. With the introduction of FNAC in suspicious lymph nodes in women who were examined during follow-up, we wanted to offer the least invasive tissue sampling method with a justifiable cost-benefit ratio. Furthermore, FNAC could be easily implemented in our follow-up procedure on the same day of the follow-up exam without major side effects. This is in accordance with various studies investigating FNAC in the primary diagnosis and staging of breast cancer [10, 16–18, 20].

A total of 2 of 38 performed FNAC probe material gathered by fine-needle aspiration were non-evaluable during cytology because insufficient cell material was obtained. Both lymph nodes were completely resected and did not reveal metastatic infiltration in specimen pathology. All 15 patients demonstrating a negative FNAC were followed up clinically. Post-interventional examinations were performed one week (physical examination) and 3 months (physical examination, ultrasonography) after FNAC in addition to the routine follow-up protocol. During our surveillance period over 22–53 months, none of the 15 patients developed lymph node recurrence in the lymph region in which FNAC was performed. In our study, no complications were observed in any of the patients who underwent FNAC, which again demonstrates the safety of FNAC [15–18, 26, 33].

Clinical examination of lymph nodes in newly diagnosed breast cancer is already difficult because large reactive lymph nodes can be misleading and small metastatic lymph nodes are not palpable [14]. Treated lymph node stations are even more difficult to examine because of possible post-treatment side effects like scarring or hematoma. This follows from our evaluation, as clinical examination was suspicious only in 11/21 malignant lymph nodes.

Though routine clinical examination is highly recommended in all follow-up programs, it is still unclear whether it contributes to relevant early recurrence detection. In a meta-analysis, there was no evidence that clinical examination contributes to survival advantage, as the majority of in-breast relapses are detected by patients or by mammography [34]. The rare axillary relapse is more often detected by clinical examination, as the axillary region is insufficiently evaluable on mammography and sonography was not performed in these studies.

Regarding sonography, all lymph nodes that were included in the study had at least one suspicious feature and only 21/37 lymph nodes were malignant. If evaluated separately, the different sonomorphological malignancy criteria reached a sensitivity and specificity ranging from 19–71 % and from 29–88 %.

Limitations of the study are the small number of patients and the single institutional execution, however the initial experiences are promising.

Another limitation of the study is that the FNAC-negative lymph nodes were not histologically verified and were only followed up. A follow-up time of at least 22 months seemed to be appropriate to define those lymph nodes as stable and consequently negative.

Sonography of lymph nodes after breast cancer treatment can be challenging. The sonographer has to differentiate between postsurgical alteration and chronic inflammation due to lymph edema or recurrent disease in the lymph nodes. The small number of patients seemed inadequate for the synthesis of further conclusions as to the value of the different ultrasound criteria.

We did not investigate the role of color Doppler ultrasound findings, which were described to be helpful in the differentiation of benign and malignant lymph nodes in primary breast cancer. Several studies have evaluated the vascularity of benign and malignant axillary lymph nodes, but the results were not consistent across the different studies, especially for lymph nodes smaller than 1 cm [35–37]. Recently, elastography was introduced as an additional feature in sonography of breast disease. In a prospective study of 104 axillary lymph nodes, strain elastography showed no improvement in diagnostic accuracy [38].

To date, it remains unclear which follow-up strategy is best for women to detect early recurrent disease and to minimize anxiety due to medical appointments. Debate persists with respect to the timeframe, extent of clinical and imaging examinations. Yearly mammography and clinical examination are the minimum guideline recommendations [11, 12, 22, 39]. Individual risk adapted follow-up examinations in future guidelines designed for optimal disease management are proposed [39, 40]. Based on the results of this study, we can recommend FNAC as an additional diagnostic step regardless of the modality of a suspicious lymph node diagnosed during follow-up exams.

## Conclusions

FNAC is a safe and reliable diagnostic tool for the evaluation of suspicious lymph nodes in breast cancer follow-up. It distinctly improves diagnostic performance of the evaluation of suspicious lymph nodes compared to clinical examination and an analysis based on sonomorphological malignancy criteria alone.

### Ethical approval and consent

This single-centre investigation was approved by the institutional review board of the University Hospital Erlangen and all procedures were in accordance with the Helsinki Declaration. The need for informed consent was waived.

## Abbreviations

FNAC: fine-needle aspiration cytology
NPV: negative predictive value
PPV: positive predictive value
